# Femoral revision knee Arthroplasty with Metaphyseal sleeves: the use of a stem is not mandatory of a structural point of view

**DOI:** 10.1186/s40634-020-00242-w

**Published:** 2020-04-26

**Authors:** F. Fonseca, A. Sousa, A. Completo

**Affiliations:** 1 Orthopaedic Surgery Department, Coimbra University Hospitals, Coimbra, Portugal; 2 Mechanical Engineering Department, University of Aveiro, 3810-193 Aveiro, Portugal

**Keywords:** Experimental strains, Finite element model, Metaphyseal sleeve, Stress-shielding, Total knee arthroplasty, Revision, Metaphyseal bony defects

## Abstract

**Purpose:**

Although metaphyseal sleeves are usually used with stems, little is known about the exact contribution/need of the stem for the initial sleeve-bone interface stability, particularly in the femur, if the intramedullary canal is deformed or bowed. The aim of the present study is (1) to determine the contribution of the diaphyseal-stem on sleeve-femur interface stability and (2) to determine experimentally the strain shielding effect on the metaphyseal femur with and without diaphyseal-stem. It is hypothesised that diaphyseal-stem addition increases the sleeve-femur interface stability and the strain-shielding effect on the metaphyseal femur relatively to the stemless condition.

**Material and methods:**

The study was developed through a combined experimental and finite-element analysis approach. Five synthetic femurs were used to measure cortex strain (triaxial-rosette-gages) behaviour and implant cortex micromotions (Digital Image Correlation) for three techniques: only femoral-component, stemless-sleeve and stemmed-sleeve. Paired t-tests were performed to evaluate the statistical significance of the difference of cortex strains and micromotions. Finite-element models were developed to assess the cancellous bone strain behaviour and sleeve-bone interface micromotions; these models were validated against the measurements.

**Results:**

Cortex strains are significantly reduced (*p* < 0.05) on the stemmed-sleeve with a 150 μstrain mean reduction at the medial and lateral distal sides which compares with a 60 μstrain mean reduction (*p* > 0.05) on the stemless condition. Both techniques presented a mean cancellous bone strain reduction of 700 μstrain (50%) at the distal region and a mean increase of 2500 μstrain (4x) at the sleeve proximal region relative to the model only with the femoral component. Both techniques presented sleeve-bone micromotions amplitude below 50-150 μm, suitable for bone ingrowth.

**Conclusions:**

The use of a supplemental diaphyseal-stem potentiates the risk of cortex bone resorption as compared to the stemless-sleeve condition; however, the stem is not essential for the enhancement of the initial sleeve-bone stability and has minor effect on the cancellous bone strain behaviour. Based on a purely structural point view, it appears that the use of a diaphyseal-femoral-stem with the metaphyseal sleeve is not mandatory in the revision TKA, which is particularly relevant in cases where the use of stems is impracticable.

## Introduction

In revision TKA (Total Knee Arthroplasty), the integrity of the remaining bone stock, once the primary components have been removed, often presents a challenge to obtain durable long-term fixation of the revision components. In these scenarios, the metaphyseal region of the bone has been recognized by its importance to the overall stability of a revision construct [15, 25]. The reconstructive techniques, including bone allograft, morselized allograft, prosthetic composites, and custom prostheses have been used, with conflicting clinical results [18, 21, 27]. With these techniques, the metaphyseal region has been underutilized, as stability is typically achieved in the epiphysis and diaphysis. Recently, metaphyseal sleeves have gained popularity as an option for patients with severe metaphyseal bony defects requiring revision TKA [9, 17]. Metaphyseal sleeves function as prosthetic structural grafts, as they allow the transfer of load from the revision components to the metaphyseal region. The potential for bony biologic fixation is a substantial benefit, when considering the use of metaphyseal sleeves. Initial sleeve stability is often achieved with use of diaphyseal-stems [1–3, 12, 20, 22, 31, 32], though few clinicians had used sleeves without stems [13, 28]. Currently, there is no consensus whether to use diaphyseal stems with metaphyseal sleeves or not [17]. Short and mid-term results have been promising; however, there are no long-term studies concerning durability [33]. The clinical results remain encouraging, but little is known about the exact structural contribution of the diaphyseal-stem for the initial sleeve-bone stability, particularly in cases where the use of stems is impracticable as bowed femoral intramedullary canals. Moreover, the stemless condition contributes to simplify the bone preparation thereby reducing operating time and reduces the revision cost. Construct stability is an important factor for the extent of biologic incorporation in the sleeve, thus enhancing the longevity of the revision procedure; however, the use of massive metal components as the sleeve and the stem changes the strain-stress bone behaviour. The purpose of the present study is (1) to determine the contribution of the diaphyseal-stem on sleeve-femur interface stability and (2) to determine experimentally the strain-shielding effect on the metaphyseal femur with and without diaphyseal-stem. It is hypothesised that diaphyseal-stem addition increases the sleeve-femur interface stability and the strain-shielding effect on the metaphyseal femur relatively to the stemless condition.

## Methods

### Experimental model

**Fig. 1:**
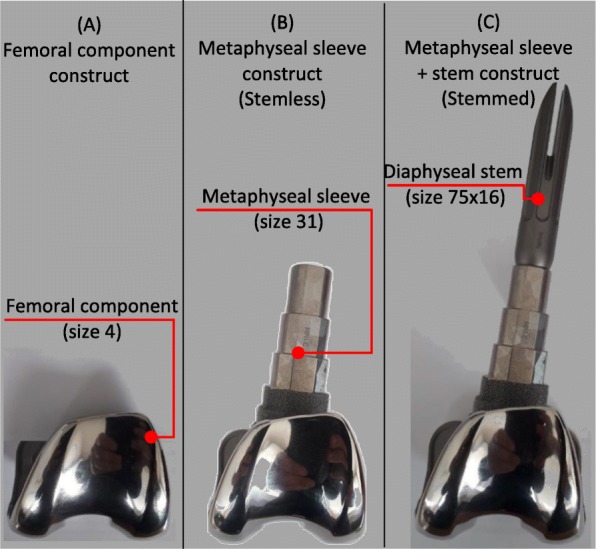
Construct techniques: (**a**) only femoral component, (**b**) femoral component with the sleeve (stemless) and (**c**) femoral component with the sleeve fastened on the diaphyseal stem (stemmed)

**Fig. 2:**
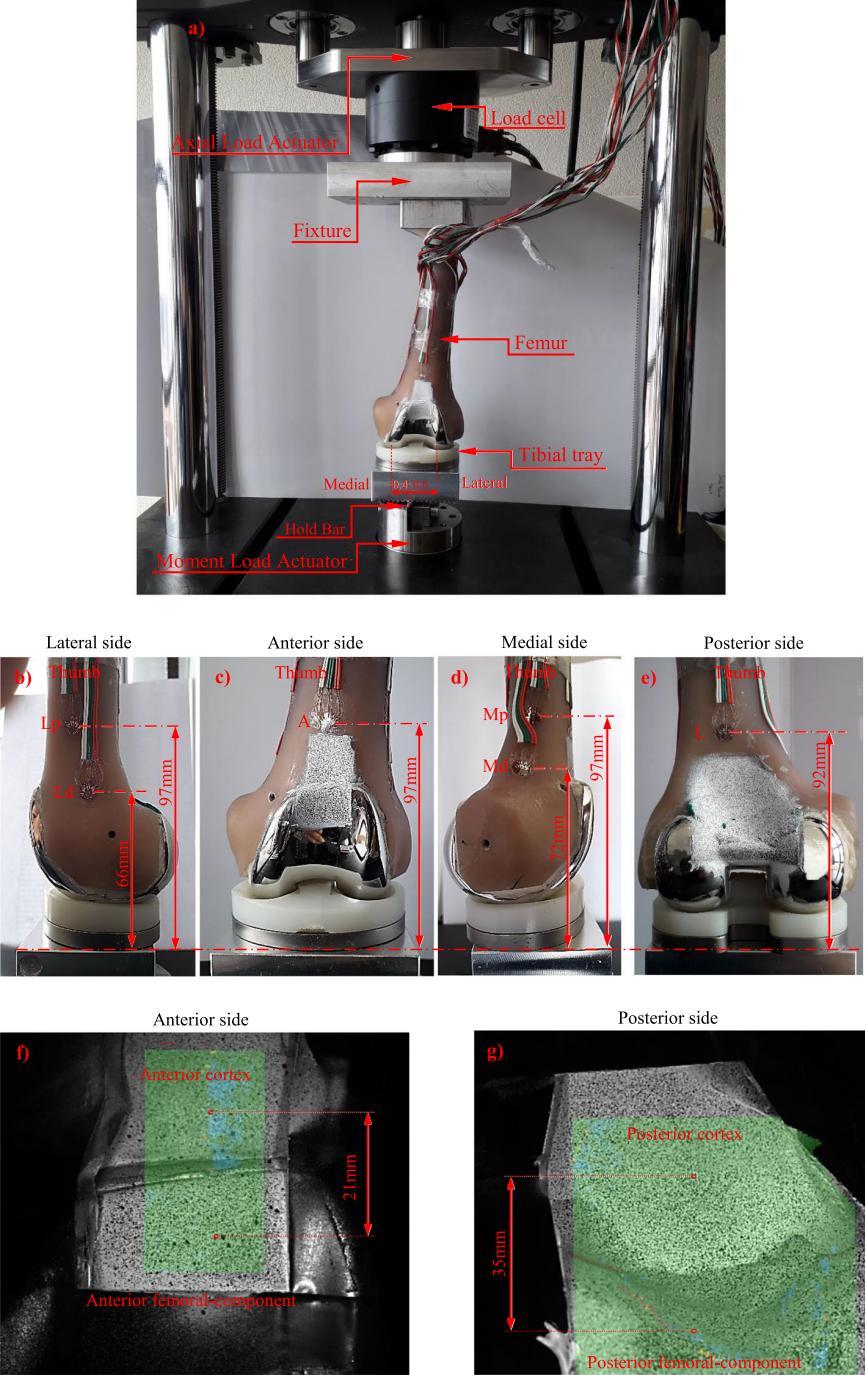
**a** Loading machine and experimental setup; **b** Strain gauges locations at Lateral side Lp (lateral proximal) and Ld (lateral distal); **c** Strain gauge location at Anterior side (A); **d** Strain gauges locations at Medial side Mp (medial proximal) and Md (medial distal); **e** Strain gauge location at Posterior side (P); **f** DIC image - stochastic pattern at Anterior femur side; g) DIC image - stochastic pattern at Posterior femur side

The study was developed through a combined experimental and finite-element modelling approach. Experimental models were developed to measure cortical strains and femoral-component/cortical-bone micromovements. Finite-element models were developed to evaluate cancellous bone strains and sleeve-cancellous bone micromovements and they were validated against measured strains and micromovements. In what concerns the experiments, five synthetic femurs (4th generation, left, mod. 3406, from Pacific Research Labs, Vashon Island, WA, USA) were selected. The physical structure of this type of synthetic bone presented stiffness and strains close to the ones measured with natural bones, exhibiting extremely low specimen-to-specimen variability [16]. The metaphyseal bone defect analysed in the present study simulates a clinical scenario of a contained-bone-defect with a volume identical to the applied metaphyseal sleeve. Each femur was tested with three different construct techniques: first only with the femoral component (A), then with the sleeve (B) and finally with the sleeve fastened with the diaphyseal stem (C) (Fig. 1). After each test, the construct and cement were carefully removed to avoid femur damage to perform subsequent construct configuration. The preparation of femoral diaphysis begins with intramedullary femoral alignment, reaming of the medullary canal until a firm endosteal engagement is reached, sequential broaching of the metaphysis to the sleeve size, femoral distal cuts, notch resection and finally femoral trial assembly. Left femoral-component TC3 (size 4), metaphyseal sleeve (size 31) and the diaphyseal-fluted-stem (size 75 mm × 16 mm) of the P.F.C Sigma Knee System (DePuy-International, Johnson&Johnson–Warsaw, USA) were all connected through the femoral adapter bolt (neutral position) and the femoral adapter-to-femoral component (7 degrees valgus angle) (Fig. 1). Only the femoral component was cemented (CMW-1) at the distal cut surfaces with a mean thickness of 2 mm (Fig. 2), which is the most common practice in revision with metaphyseal-sleeves.

### Testing protocol

Six triaxial strain gauges (KFG-3-120-D17-11L3M2S Kyowa-Electronic-Instruments, Japan) were glued to the distal femur at the medial (Md, Mp), lateral (Ld, Lp), anterior (A) and posterior (P) sides. The strain gauges positions were chosen in order to be located in the metaphyseal sleeve region (Fig. 2b, c, d and e). All strain gauges were connected to a data acquisition system PXI-1050 (National-Instruments, USA). Three load-cases (in extension) were applied experimentally. First, an axial load of 2030 N (3x bodyweight) was applied in the mid-shaft of the femur with the femoral-component in contact with the tibial tray (TestRessouces Axial-Torsion Test Machine, MN, USA), which due to the hold bar placed medially under the tibial tray support, a load repartition of 60% and 40% at the medial and lateral condyle respectively was guaranteed (Fig. 2a). The second load-case was a pure internal-external moment of 7 Nm applied through the tibial tray to the femoral-component. The tibial tray support was fastened to the bench of the angle/moment actuator of the loading machine (Fig. 2). The third load-case was the combination of the two previous load-cases, where simultaneously the axial load of 2030 N and the internal-external moment of 7 Nm were applied. These applied loads are representative of a normal physiological loading condition during walking at the stance phase before toe-off [24]. To correlate with finite-element models and evaluate the risk of strain-shielding at the metaphyseal cortex region, the maximum (ε1) and minimum (ε2) principal-strains within the plane of the gauge were calculated and averaged. The femoral-component total displacement (micromotion) relative to the anterior and posterior femoral cortex, was measured after 100 load cycles at a frequency of 1 Hz, for each construct configuration, using the commercial DIC (Digital image correlation) system ARAMIS 5 M (GOM Precise Industrial 3D Metrology, Germany). Images were acquired using Photron APX—RS high-speed cameras having a 2448 × 2050 pixel sensor along with 105 mm Fixed Focal Length Nikon lenses, pointed to the anterior and posterior femur region at 200 mm. The field view was set to 50 mm (width) by 50 mm (height), with a depth field of 20 mm. This volume is enough to frame the entire region of interest (ROI), first the anterior and then the posterior femur cortex. At the start of each test, a rigid calibration target was first moved in the location where the femur would be positioned for calibration of the DIC images. Images were taken every 0.1 s for the duration of the tests. ARAMIS v6.2.0–6 software was used to measure pixel displacement, and thus calculate the total relative displacements (micromotion) between the anterior cortex and the anterior femoral-component (a distance of 21 mm) and between the posterior cortex and the posterior femoral-component (a distance of 35 mm) (Fig. 2f and g).

### Finite-element analysis

Finite-element (FE) models of the three implanted configurations were built from radiographs and CT-scans of the experimental models. Models meshes and non-linear analyses were performed with ABAQUS (Abaqus 2017, Simulia, Providence, USA). The cement-implant and implant-bone interfaces were modelled with a surface-to-surface contact algorithm using coefficients of friction of 0.25 and 0.3 [5] respectively. The bone-cement was considered rigidly bonded to the bone. The convergence rate of the maximum displacement of the FE models for more than 180,000 tetrahedral elements was less than 0.5%, in all models. The materials were assumed to be homogeneous, isotropic and linearly elastic; the elastic modulus values adopted for the femoral component, sleeve and fluted stem, cement, cortical and cancellous bone were 210 GPa, 110 GPa, 16.7 GPa and 0.155 GPa, respectively [5, 6]. Poisson’s ratio was considered to be 0.3 for all materials [5, 6]. The three load-cases applied to the FE models replicates those used in the experimental setup. Principal bone strains acting on the gauge planes were selected corresponding to the experimental strain measurement sites. Regression analyses of the principal strains predicted by the FE models and measured strains were performed. The root-mean-square-error, expressed as a percentage (RMSE %) of the peak values of the measured principal-strains, was used as an additional indicator of the overall absolute difference between numerical and experimental strains. The relative FE total displacement (micromotion) between the anterior and posterior femur cortex the femoral-component were compared with the experimental ones. To evaluate, cancellous bone failure risk in compression around the metaphyseal sleeve, comparative analyses of the minimal-principal-strains were conducted for each construct. Finally, the micro-movements between the metaphyseal-sleeve and the cancellous bone at the anterior, posterior, medial and lateral contact areas were evaluated.

### Statistical analysis

**Fig. 3:**
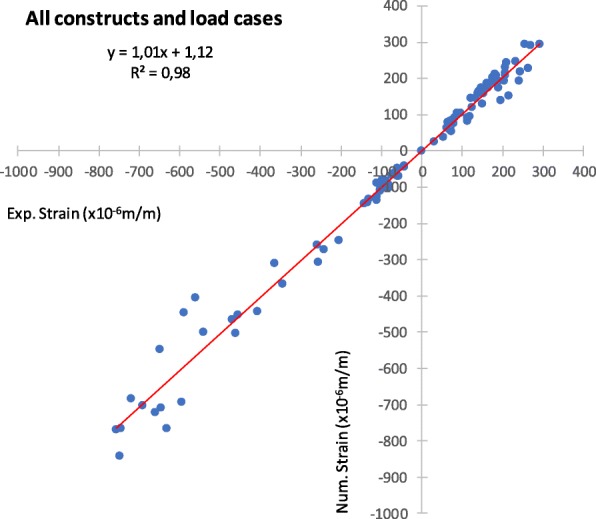
Linear regression between experimental and numerical cortical strains (all strain gauges, all construct techniques and load cases)

An exploratory data analysis was made to check the normal distribution of all data. Paired t-tests were performed (SPSS, USA) to evaluate the statistical significance of the difference between mean principal strains and implant-cortex micromotions. Statistically, significant differences are considered for *p*-values lower than 0.05. The sample size was based on the estimation of the standard deviation from previous identical studies [5–8] for a α = 0.05 and a power of 0.8. The linear regression between all measured and finite-element principal cortex strains presented a correlation value (R^2^) of 0.98 and a slope of 1.01 (Fig. 3).

## Results

### Experimental

**Fig. 4:**
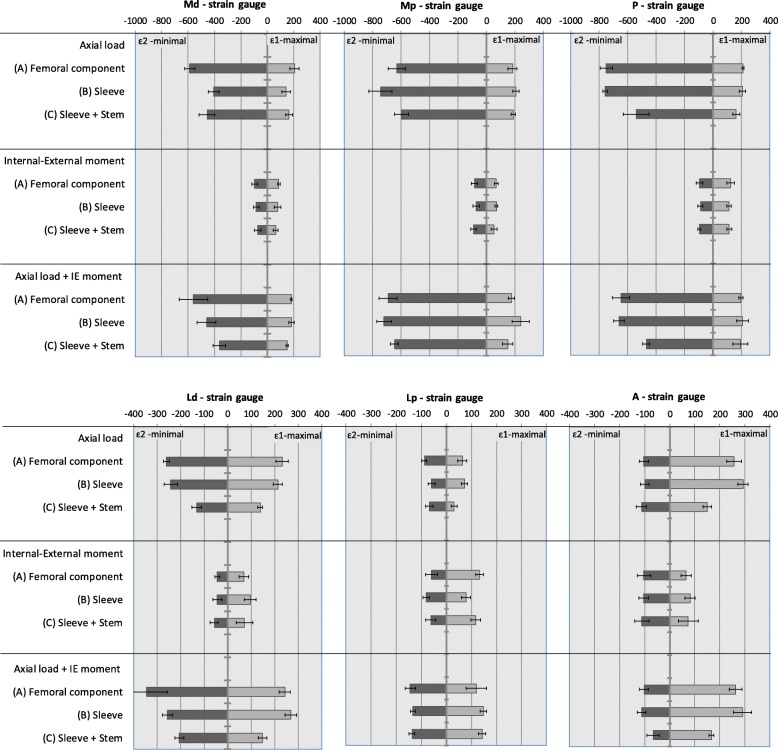
Mean and standard deviation (error bars) of the measured principal strains (ε1 - maximal and ε2 - minimal) at each strain gauge Md (medial distal), Mp (medial proximal), P (posterior), A (anterior), Ld (lateral distal) and Lp (lateral proximal) for each construct technique and load case

The means and standard deviations of cortex principal-strains at each strain gauge are presented in Fig. 4 for all load-cases. The average standard deviation of the principal-strains was less than 11% (Fig. 4). The Internal-External moment load case presents the lowest principal-strains of the three load-cases analysed with a nominal mean value below 150μstrain. The Axial and Axial + Internal-External moment load cases presented very similar cortex strain behaviour. On these load cases, the highest nominal minimum-principal-strains (compressive) were recorded at the medial (Md, Mp) and posterior (P) strain gauges, while the highest maximum-principal-strains (tensile) were measured at the lateral (Ld, Lp) and anterior (A) strain gauges. Excluding the Anterior (A) strain gauge, the magnitude of minimum-principal-strains was greater than maximum-principal-strains, with nominal values of 750 μstrain measured at the Posterior (P) and Medial-proximal (Mp) strain gauges. Significant principal cortex strain changes (*p* < 0.05) between the three different techniques were observed mainly in the Axial and Axial + Internal-External moment load cases (Table 1). For these two load cases, only two strain gauges (33%) presented significant principal strains changes between the techniques with femoral component (A) and stemless-sleeve (B). In contrast, a significant principal strain reduction in five strain-gauges (83%) is present between the techniques with femoral component (A) and stemmed-sleeve (C).

**Table 1:** *P*-values obtained from T-tests to test differences between the means of the minimal (ε2) and the maximal (ε1) principal strains. For a level of significance α, statistically significant differences will be detected when *p*-value< 0.05

**Load case**	**Strain gauge**		**Femoral component (A) versus Sleeve (B)**		**Femoral component (A) versus Sleeve + Stem (C)**	
			**ɛ2 (minimal)**	**ɛ1 (maximal)**	**ɛ2 (minimal)**	**ɛ1 (maximal)**
**Axial load**	Medial distal	Md	0,002 (*P* < 0.01)	0,02 (*P* < 0.05)	0,004 (P < 0.01)	0,005 (*P* < 0.01)
	Medial proximal	Mp	NS	NS	NS	NS
	Posterior	P	NS	NS	0,008 (*P* < 0.01)	0,01 (*P* < 0.05)
	Lateral distal	Ld	NS	NS	0,001 (*P* < 0.01)	0,001 (*P* < 0.01)
	Lateral proximal	Lp	0,01 (P < 0.05)	NS	0,02 (*P* < 0.05)	0,004 (*P* < 0.01)
	Anterior	A	NS	NS	NS	0,001 (*P* < 0.01)
**Internal-External moment**	Medial distal	Md	NS	NS	NS	0,01 (*P* < 0.05)
	Medial proximal	Mp	NS	NS	NS	NS
	Posterior	P	NS	NS	NS	NS
	Lateral distal	Ld	NS	NS	NS	NS
	Lateral proximal	Lp	0,02 (P < 0.05)	0,001 (P < 0.01)	NS	NS
	Anterior	A	NS	NS	NS	NS
**Axial load + Internal-External moment**	Medial distal	Md	0,01 (P < 0.05)	NS	0,002 (*P* < 0.01)	0,003 (*P* < 0.01)
	Medial proximal	Mp	NS	0,04 (P < 0,05)	0,04 (*P* < 0.05)	NS
	Posterior	P	NS	NS	0,002 (*P* < 0.01)	NS
	Lateral distal	Ld	NS	NS	0,02 (*P* < 0.05)	0,002 (*P* < 0.01)
	Lateral proximal	Lp	NS	NS	NS	NS
	Anterior	A	NS	NS	0,04 (*P* < 0.05)	0,001 (*P* < 0.01)

The measured micromotions (DIC) between femoral-component and anterior and posterior cortex are presented in Table 2. No statistically significant micromotion differences were found between the techniques stemless-sleeve (B) vs. femoral component (A) and the stemmed-sleeve (C) vs. femoral component (A) for all load cases.

**Table 2:** Mean and standard deviation (SD) of the measured total micromotion between the femoral-component and the anterior and posterior femoral cortex for the different reconstructive techniques and load cases. P-values obtained from T-tests to test differences between means of the measured micromotions. Finite element (FE) model total micromotion results and relative difference (Dif.) to the measured

Load case	Cortex side	Experimental (DIC)								Finite element model					
		Total micromotion (μm)						P - values		Total micromotion (μm)					
		Femoral component (A)		Sleeve (B)		Sleeve + Stem (C)		Femoral component (A) versus Sleeve (B)	Femoral component (A) versus Sleeve + Stem (C)	Femoral component (A)		Sleeve (B)		Sleeve + Stem (C)	
		Mean	SD	Mean	SD	Mean	SD			Value	Dif. FE-DIC	Value	Error FE-DIC	Value	Dif. FE-DIC
Axial load	Anterior	10	6	7	3	7	3	NS	NS	11	+ 1	9	+ 2	8	+ 1
	Posterior	39	12	35	8	32	5	NS	NS	42	+ 3	38	+ 3	30	−2
Internal-External moment	Anterior	12	6	9	4	8	3	NS	NS	10	−2	7	-2	9	+ 1
	Posterior	10	3	7	3	8	3	NS	NS	12	+ 2	9	+ 2	7	−1
Axial load + Internal-External moment	Anterior	11	4	11	5	14	4	NS	NS	9	+ 2	10	−1	8	−6
	Posterior	32	9	28	9	32	8	NS	NS	37	+ 5	33	+ 1	29	−7

### Finite-element

The overall absolute difference between finite-element and experimental cortex strains (RMSE %) was 11%. The difference between finite-element and experimental implant-cortex micromotion ranged between -7 μm and + 5 μm (Table 2), which represents a mean difference of 15%.

**Fig. 5:**
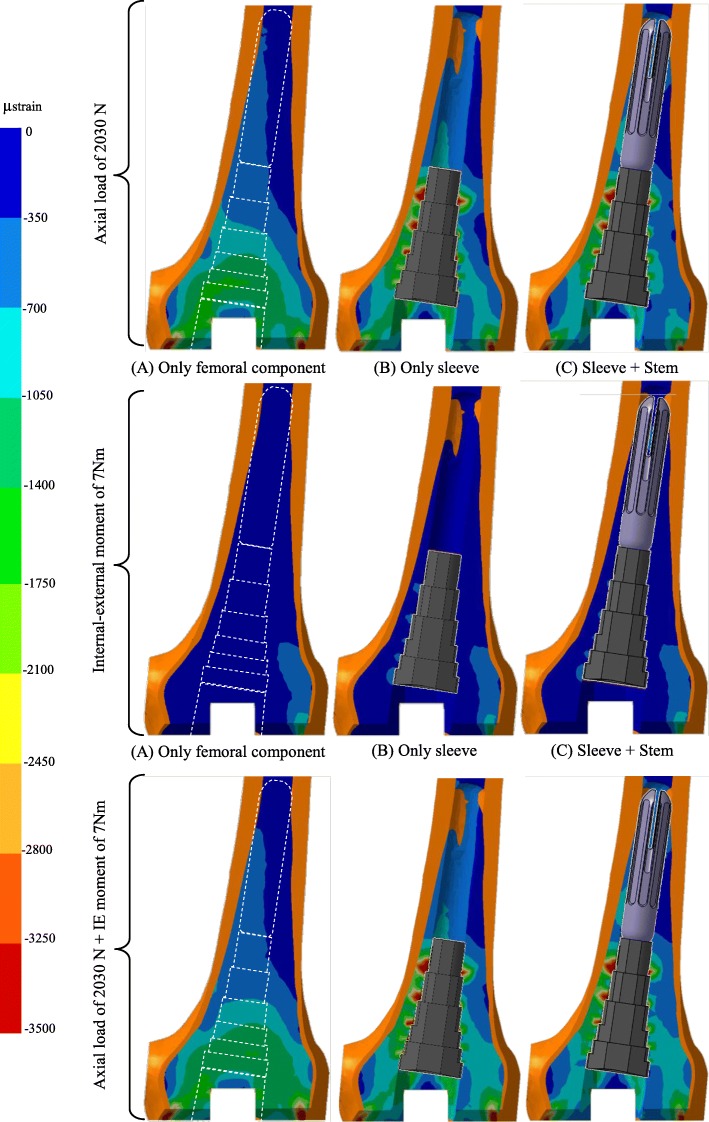
Minimal principal strains in cancellous bone for the axial load case (upper row), internal-external moment load case (middle row) and axial load + internal-external moment load case (lower row) on the construct technique only with femoral component (left column), with sleeve (centre column) and with sleeve and stem (right column). The colour gradient specifies the range of principal strains

Figure 5 shows the patterns of the minimum-principal-strains in cancellous bone obtained in all FE analyses. The Internal-External moment load case has the lowest minimum-principal-strains in cancellous bone of all load cases, with nominal mean values below -700μstrain at the sleeve region. Very similar cancellous bone strain behaviour is present in the Axial and Axial + IE moment load cases. The highest nominal minimum-principal-strains in cancellous bone were reached at the sleeve proximal region with peak values about -3250μstrain, for both techniques stemless-sleeve (B) and stemmed-sleeve (C). These two techniques reduce about 50% the cancellous bone strain at the distal femur region (femoral-component) and an increase nearly four times at the proximal metaphyseal region around sleeve comparing to the model only with femoral component (A).

Micromotion on the cancellous bone-sleeve interface on the anterior, medial, lateral and posterior femur sides is presented in Table 3. The lowest micromotions, below 10 μm, were registered on the Internal-External moment load case, while the highest mean micromotions occurred in the Axial and Axial + IE moment load cases. For these two load cases, the lowest micromovements happened at the sleeve distal region, with values below the 16 μm, while at the proximal sleeve region were registered the peak values that ranged between the 28 μm and 70 μm for both techniques stemless-sleeve (B) and stemmed-sleeve (C). No substantive micromotion differences were found between the different sleeve sides for all models. The diaphyseal-stem addition (C) reduces the micromotions in all sleeve-bone interface sides relative to the stemless-sleeve technique (B), these reductions are more important at the proximal sleeve region with a mean value of 42%, while at the sleeve distal region these reductions were 20%.

**Table 3:** Interface micromotions (μm) between sleeve and cancellous-bone along medial, anterior, lateral and posterior sides

**Medial**							**Anterior**						
**Load case**	**Axial**		**IE moment**		**Axial + IE moment**		**Load case**	**Axial**		**IE moment**		**Axial + IE moment**	
(μm)	Only Sleeve	Sleeve + stem	Only Sleeve	Sleeve + stem	Only Sleeve	Sleeve + stem	(μm)	Only Sleeve	Sleeve + stem	Only Sleeve	Sleeve + stem	Only Sleeve	Sleeve + stem
	41	34	6	5	45	34		54	29	4	4	60	33
	30	28	6	5	32	29		35	24	4	3	39	25
	23	21	5	5	25	23		20	18	3	3	22	19
	20	19	5	4	23	20		18	15	3	3	21	17
	17	16	4	3	19	17		16	13	3	2	18	14
	14	15	4	3	16	14		14	12	3	2	16	10
**Lateral**							**Posterior**						
**Load case**	**Axial**		**IE moment**		**Axial + IE moment**		**Load case**	**Axial**		**IE moment**		**Axial + IE moment**	
(μm)	Only Sleeve	Sleeve + stem	Only Sleeve	Sleeve + stem	Only Sleeve	Sleeve + stem	(μm)	Only Sleeve	Sleeve + stem	Only Sleeve	Sleeve + stem	Only Sleeve	Sleeve + stem
	64	38	5	5	70	40		48	25	10	8	52	28
	40	32	5	4	43	34		35	22	8	7	37	25
	30	25	4	4	33	26		27	19	7	7	29	20
	20	18	3	3	22	19		22	17	5	4	23	18
	16	15	3	2	17	15		18	14	4	4	19	14
	12	10	2	2	12	9		12	11	3	3	14	9

## Discussion

It was hypothesised that diaphyseal-stem addition increases the sleeve-femur interface stability and the strain-shielding effect on the metaphyseal femur in relation to the stemless condition. The obtained results to some extent contradict the study hypothesis. The use of a supplemental diaphyseal-stem potentiates the risk of cortex bone resorption compared with the stemless-sleeve condition; however, the stem is not essential to increase the initial sleeve-bone stability and has a minor effect on the cancellous bone strain behaviour. To the authors’ knowledge, there are no other studies that had evaluated the contribution of the diaphyseal-stem for the initial sleeve-femur construct stability, as well as, the metaphyseal femur strain behaviour neither in-vitro nor using the FE method. The standard deviations of the measured cortex strains were within the range of those found in the literature which used the synthetic bone models [6, 23]. The principal cortex strains behaviour for the Axial and Axial + Internal-External moment load cases were nearly identical, with nominal minimum-principal-strains values, on average, 4 to 8 times greater than the Internal-External moment load case. These cortex strain differences, between load cases, are related with the great magnitude of the axial load component and their asymmetric distribution between the medial and lateral condyles on the tibial tray, which induce a frontal moment and thus high compressive and tensile strains at medial and lateral femur sides, respectively. The experimental cortex strain results demonstrate that metaphyseal femur cortex is apparently immune to the presence of the sleeve (B) or the sleeve + stem (C) when only subjected to an Internal-External moment load. However, when the axial load component is present the cortex strains are significantly reduced (*p* < 0.05) in 83% of strain gauges on stemmed-sleeve, which compares with 33% in stemless condition, relative to the femoral component alone (A). It is known that in situations where bone loads are reduced or eliminated, bone mass is reabsorbed [14]. However, the nominal metaphyseal cortex strain reduction was inferior to 50 − 200μstrain in most of the strain gauges, whereby seem to present a limited risk of change of the cortex remodelling process [10], i.e. not enough to reduce cortex bone density when compared with the femoral component alone (A).Overall, the stemmed-sleeve technique (C) increased the femoral component stability relative to the anterior and posterior metaphyseal cortex when compared with the stemless-sleeve technique (B); however, no significant micromotions differences were found, when compared with the femoral component alone (A).

The FE models developed to assess the structural behaviour of cancellous bone presented a good correlation between numerical and experimental cortex strains in the range of previously published studies [7, 8], as well as implant-cortex stability presented reduced micromotion differences between numerical and experimental, which demonstrates the reliability of the FE models. The critical factor to the bone structure is the risk of failure of the supporting cancellous-bone in compression [4, 30]. The failure process of cancellous bone can be due to overload, and usually, it is a fatigue mode or failure described by Wolff’s law; in situations where bone loads are reduced or eliminated, bone mass is reabsorbed [14]. When the axial load component is present, both techniques (B and C) presented identical peak minimum-principal cancellous bone strains values at the sleeve proximal region, increasing nearly four times relative to the model only with femoral component (A), while both techniques reduce cancellous bone strain at the distal femur region. The load transfer effect of the sleeve in both techniques increases the risk of proximal metaphyseal cancellous bone to suffer fatigue failure. It is reasonable to expect cancellous bone to suffer fatigue failure, when the number of cycles is greater than 1 million [30] if the induced strains are increased by 50 to 100% due to implantation [4], which is the present case. To reduce this risk, the limitation of the patient weight-bearing immediately after the revision with the sleeve can lead to the positive outcome of the procedure by reducing the overload effect at the proximal metaphyseal region. As previously mentioned, an important factor on the sleeve cancellous bone osseointegration is the interface micromotion amplitude [26, 29]. The amplitude of micromotions should be < 150 μm to achieve good osseointegration, higher amplitudes of the micromotions leads to the formation of fibrous tissue and future implant loosening [19]. The diaphyseal-stem addition (C) reduces the distal sleeve-bone interface micromotion; however, the maximum micromotion values on the stemless-sleeve technique (B) at the distal sleeve region (porous coated surfaces) were inferior to 33 μm. Given the obtained results, the average amplitude of micromotions for both techniques lies below the aforementioned critical limit of 150 μm. Therefore, it can be concluded that the general mechanical performance of both techniques, stemless and stemmed, are suitable for bone ingrowth.

The present study has limitations. The first one is related to the use of synthetic femur and to simplifications in the experiments to represent the functioning knee after revision with a metaphyseal femoral sleeve. However, the flexural and torsional rigidity of synthetic femur is within the range of values verified for healthy adult bones; also the failure modes of the synthetic models are close to published data for human bones [11]. Another limitation is the simplified loads applied, although they are representative of the major loads acting upon the implant and femur structure. In addition, this study does not account for the associated thinning, quality of the cortices, loss of density in the adjacent cancellous bone and different metaphyseal bone defect geometries. In an in vivo situation these parameters will affect the load share and the implant stability; even so, it seems reasonable to expect that these conditions will affect in the same way the different techniques. Even though, the goal of this study was to gain understanding on how the two techniques applied in identical femur conditions can be associated with a life expectancy of the revision procedure; this insight will lead to an improved surgical decision process, which will be based on independent scientific understanding and advanced prediction tools.

## Conclusions

The use of a supplemental diaphyseal-stem may increase the risk of cortex bone resorption compared with the stemless-sleeve condition; moreover, the stem is not essential to enhance the initial sleeve-bone stability. Both techniques are suitable for sleeve-bone ingrowth, and the diaphyseal-stem also, has minor effect on the cancellous bone strain behaviour; the limit on patient weight-bearing after the revision procedure can contributes to the reduction of the overload effect at the proximal sleeve region. Based on a purely structural point view, it appears that the use of a diaphyseal-femoral-stem with the metaphyseal sleeve is not mandatory in the revision. These findings do not fully support the study original hypothesis.
